# *catena*-Poly[[[{5,5′-dimeth­oxy-2,2′-[ethane-1,2-diylbis(nitrilo­methyl­idyne)]diphenolato}manganese(III)]-μ-acetato] methanol monosolvate]

**DOI:** 10.1107/S160053681003984X

**Published:** 2010-10-09

**Authors:** Gervas E. Assey, Anand M. Butcher, Ray J. Butcher, Yilma Gultneh

**Affiliations:** a Department of Chemistry, Howard University, 525 College Street NW, Washington, DC 20059, USA

## Abstract

The title Mn^III^ compound, {[Mn(C_18_H_18_N_2_O_4_)(CH_3_COO)]·CH_3_OH}_*n*_, was synthesized by a reaction between mangan­ese(II) acetate and ethyl­enebis(4-meth­oxy­salicylaldimine). The structure is made up of bis­(4-meth­oxy­salicyldene)ethyl­enediaminatomanganese(III) units bridged by acetate groups, with Mn—N = 1.9786 (9), Mn—O = 1.8784 (10) and Mn—O_acetate_ = 2.056 (9) and 2.2571 (9) Å, forming a one dimensional polymer (–Mn–acetate–Mn–acetate–) along [100]. The Mn^III^ atom is in a Jahn–Teller-distorted octa­hedral environment with *cis* angles ranging from 81.87 (4) to 96.53 (4)° and *trans* angles ranging from 166.11 (3) to 173.93 (3)°. The methanol solvent mol­ecule is hydrogen bonded to the phenolate O atom. In addition to this classical hydrogen bond, there are weak C—H⋯O inter­actions. The structure was determined from a crystal twinned by pseudo-merohedry.

## Related literature

For the biological activity of manganese(III) complexes with tetra­dentate Schiff bases derived from salicyl­aldehyde, see: Watkinson *et al.* (1999 ▶); Mandal *et al.* (2009 ▶); Hulme *et al.* (1997 ▶); Suzuki *et al.* (1997 ▶); Thampidas *et al.* (2008 ▶). For the oxidation of organic compounds using transition metal catalysts, see: Jang & Jacobsen (1991 ▶); Kochi (1978 ▶).
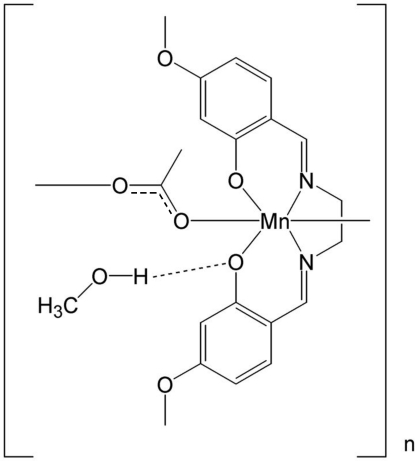


## Experimental

### 

#### Crystal data

[Mn(C_18_H_18_N_2_O_4_)(C_2_H_3_O_2_)]·CH_4_O
*M* *_r_* = 472.37
Monoclinic, 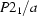

*a* = 6.6237 (2) Å
*b* = 21.5007 (6) Å
*c* = 14.5544 (4) Å
β = 97.539 (3)°
*V* = 2054.84 (10) Å^3^
*Z* = 4
Mo *K*α radiation
μ = 0.69 mm^−1^
*T* = 110 K
0.52 × 0.41 × 0.16 mm

#### Data collection

Oxford Diffraction Gemini diffractometer with Ruby detector
Absorption correction: multi-scan (*CrysAlis RED*; Oxford Diffraction, 2007 ▶) *T*_min_ = 0.914, *T*_max_ = 1.000
27951 measured reflections
27951 independent reflections
23528 reflections with *I* > 2σ(*I*)

#### Refinement

*R*[*F* ^2^ > 2σ(*F* ^2^)] = 0.050
*wR*(*F* ^2^) = 0.135
*S* = 1.03
27951 reflections
286 parameters
6 restraints
H-atom parameters constrained
Δρ_max_ = 0.85 e Å^−3^
Δρ_min_ = −0.69 e Å^−3^

### 

Data collection: *CrysAlis PRO* (Oxford Diffraction, 2007 ▶); cell refinement: *CrysAlis PRO*; data reduction: *CrysAlis PRO*; program(s) used to solve structure: *SHELXS97* (Sheldrick, 2008 ▶); program(s) used to refine structure: *SHELXL97* (Sheldrick, 2008 ▶); molecular graphics: *SHELXTL* (Sheldrick, 2008 ▶); software used to prepare material for publication: *SHELXTL*.

## Supplementary Material

Crystal structure: contains datablocks I, global. DOI: 10.1107/S160053681003984X/bt5366sup1.cif

Structure factors: contains datablocks I. DOI: 10.1107/S160053681003984X/bt5366Isup2.hkl

Additional supplementary materials:  crystallographic information; 3D view; checkCIF report

## Figures and Tables

**Table 1:** Hydrogen-bond geometry (Å, °)

*D*—H⋯*A*	*D*—H	H⋯*A*	*D*⋯*A*	*D*—H⋯*A*
O1*S*—H1*S*⋯O2	0.84	2.08	2.8484 (15)	153
C8—H8*A*⋯O11^i^	0.95	2.51	3.4152 (15)	158
C8—H8*A*⋯O12^i^	0.95	2.63	3.4802 (14)	150
C16—H16*B*⋯O3^ii^	0.98	2.52	3.0358 (17)	113

Symmetry codes: (i) 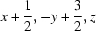; (ii) 
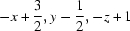
.

**Fig. 1.**
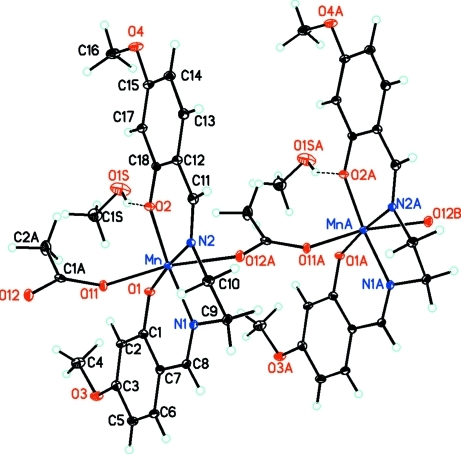
Diagram showing the bis(4-methoxysalicyldene)ethylenediaminatomanganese(III) moieties bridged by acetate groups and methanol solvate hydrogen bonded to the phenolic oxygen atoms (shown by dashed lines).

**Fig. 2.**
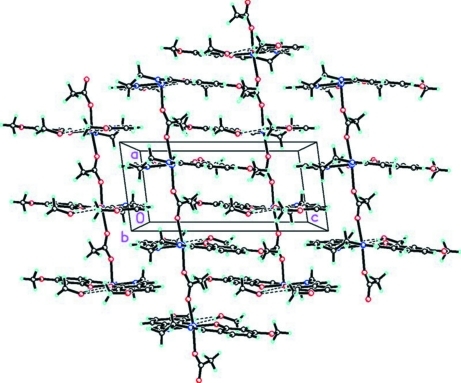
The molecular packing for C36H40ClN6Ni2O9 viewed down the b axis showing the linear chains of bis(4-methoxysalicyldene)ethylenediaminatomanganese(III) moieties bridged by acetate groups and methanol solvate hydrogen bonded to the phenolic oxygen atoms (shown by dashed lines).

## supplementary crystallographic information

### Comment

Manganese(III) complexes with tetradentate Schiff base derived from salicylaldehyde are of great importance due to their application in many biological activities (Watkinson *et al.*, 1999). These biological activities involve the multinuclear cluster of manganese ions within the oxygen evolving complex (OEC) of photosystem(II). This cluster is involved in the photolytic oxidation of water to dioxygen within the OEC of the photosystem(II) (Hulme *et al.*, 1997; Mandal *et al.*, 2009). Other biological activities where the role of manganese has played part are in enzymes such as superoxide dismutase, catalase and orginase (Thampidas *et al.* 2008). The importance of molecular oxygen to all animals on earth including man is for metabolism and to provide energy for life (Suzuki *et al.*, 1997).

Also organic compounds can be oxidized using transition metal catalysts (Kochi, 1978; Jang & Jacobsen, 1991).

In the title compound C_21_H_25_MnN_2_O_7_ the structure is made up of bis(4-methoxysalicyldene)ethylenediaminatomanganese(III) moieties bridged by acetate groups with Mn—N(1) = 1.9786 (9) Å and Mn—O(1) = 1.878 (10) Å and Mn—O_acetate_ = 2.056 (9) Å forming a one dimensional polymer (–Mn-acetate-Mn-acetate-). The Mn atom is in a distorted octahedral environment with *cis* angles ranging from 81.87 (4)° to 96.54 (4)°. Each manganese(III) ion is at the center of nearly square plane with bond lengths Mn—N(1) = 1.9786 (9) Å, Mn—N(2) = 1.9954 (11) Å, Mn—O(1) = 1.8784 (10) Å and Mn—O(2) = 1.9135 (7) Å. An axial elongation of Mn—*O*(acetate) *i.e.* Mn—O(II) = 2.2056 (9) Å is an indication of the Jahn Teller distortion which is expected for a high spin manganese(III) ion in six-coordinate environment. The methanol of solvation is hydrogen bonded to the phenolic oxygen. In addition there are weak C—H···O interactions.

The structure is made up of bis(4-methoxysalicyldene)ethylenediaminatomanganese(III) moieties bridged by acetate groups with Mn—N(1) = 1.9786 (9) Å and Mn—O(1) = 1.878 (10) Å and Mn—*O*\ãcetate\~ = 2.056 (9) Å forming a one dimensional polymer (–Mn-acetate-Mn-acetate-). The Mn atom is in a distorted octahedral environment with *cis* angles ranging from 81.87 (4)° to 96.54 (4)°. The methanol of solvation is hydrogen bonded to the phenolic oxygen. In addition there are weak C—H···O interactions.

### Experimental

The synthesis of the ligand ethylenebis(4-methoxysalicylaldimine) was achieved by the reaction of a solution of (1 g, 16.6 mmol) ethylenediamine in 20 ml me thanol with a solution of (5.0 g, 33.3 mmol) 2-hydroxy-*p*-anisaldehyde in 40 ml e thanol. This was added dropwise using glass pipette into a round bottomed flask containing the ethylene diamine. The mixture was refluxed for 24 h. Yellow solids were obtained upon solvent removal by evaporation under reduced pressure and drying.

The synthesis of the complex C_21_H_25_MnN_2_O_7_ was achieved by adding a solution of (0.33 g, 1 mmol) ethylenebis(4-methoxysalicylaldimine) in 3 ml chloroform drop wise to a solution of Mn(CH_3_COO)_2_.4H_2_O (0.25 g, 1 mmol) in 5 ml me thanol. The mixture was stirred for 1 h and then layered with diethyl ether for slow diffusion crystallization process. Crystals suitable for X-ray diffraction were obtained.

### Refinement

H atoms were placed in geometrically idealized positions and constrained to ride on their parent atoms with a C—H distance of 0.95 and 0.99 Å *U*_iso_(H) = 1.2*U*_eq_(C) and 0.98 Å for CH_3_ [*U*_iso_(H) = 1.5*U*_eq_(C)]. The H atoms attached to N were idealized with an N–H distance of 0.91 Å. One atom (C1A) did not behave well when refined anisotropically. This atom was restrained to an isotropic behavior. The crystal was twinned by a 180° rotation about the c-axis, the contribution of the minor twin component refined to 0.3809 (6).

### Crystal data

**Table:**

[Mn(C_18_H_18_N_2_O_4_)(C_2_H_3_O_2_)]·CH_4_O	*F*(000) = 984
*M**_r_* = 472.37	*D*_x_ = 1.527 Mg m^−^^3^
Monoclinic, *P*2_1_/*a*	Mo *K*α radiation, λ = 0.71073 Å
Hall symbol: -P 2yab	Cell parameters from 13341 reflections
*a* = 6.6237 (2) Å	θ = 4.7–32.6°
*b* = 21.5007 (6) Å	µ = 0.69 mm^−^^1^
*c* = 14.5544 (4) Å	*T* = 110 K
β = 97.539 (3)°	Plate, light green
*V* = 2054.84 (10) Å^3^	0.52 × 0.41 × 0.16 mm
*Z* = 4	

### Data collection

**Table:**

Oxford Diffraction Gemini diffractometer with Ruby detector	27951 independent reflections
Radiation source: Enhance (Mo) X-ray Source	23528 reflections with *I* > 2σ(*I*)
graphite	*R*_int_ = 0.0000
Detector resolution: 10.5081 pixels mm^-1^	θ_max_ = 32.7°, θ_min_ = 4.7°
ω scans	*h* = −10→8
Absorption correction: multi-scan (*CrysAlis RED*; Oxford Diffraction, 2007)	*k* = −31→31
*T*_min_ = 0.914, *T*_max_ = 1.000	*l* = −21→21
27951 measured reflections	

### Refinement

**Table:**

Refinement on *F*^2^	Primary atom site location: structure-invariant direct methods
Least-squares matrix: full	Secondary atom site location: difference Fourier map
*R*[*F*^2^ > 2σ(*F*^2^)] = 0.050	Hydrogen site location: inferred from neighbouring sites
*wR*(*F*^2^) = 0.135	H-atom parameters constrained
*S* = 1.03	*w* = 1/[σ^2^(*F*_o_^2^) + (0.0898*P*)^2^] where *P* = (*F*_o_^2^ + 2*F*_c_^2^)/3
27951 reflections	(Δ/σ)_max_ = 0.002
286 parameters	Δρ_max_ = 0.85 e Å^−^^3^
6 restraints	Δρ_min_ = −0.68 e Å^−^^3^

### Special details

**Table:**

Geometry. All e.s.d.'s (except the e.s.d. in the dihedral angle between two l.s. planes) are estimated using the full covariance matrix. The cell e.s.d.'s are taken into account individually in the estimation of e.s.d.'s in distances, angles and torsion angles; correlations between e.s.d.'s in cell parameters are only used when they are defined by crystal symmetry. An approximate (isotropic) treatment of cell e.s.d.'s is used for estimating e.s.d.'s involving l.s. planes.
Refinement. Refinement of *F*^2^ against ALL reflections. The weighted *R*-factor *wR* and goodness of fit *S* are based on *F*^2^, conventional *R*-factors *R* are based on *F*, with *F* set to zero for negative *F*^2^. The threshold expression of *F*^2^ > σ(*F*^2^) is used only for calculating *R*-factors(gt) *etc*. and is not relevant to the choice of reflections for refinement. *R*-factors based on *F*^2^ are statistically about twice as large as those based on *F*, and *R*- factors based on ALL data will be even larger.

### Fractional atomic coordinates and isotropic or equivalent isotropic displacement parameters (Å^2^)

**Table:**

	*x*	*y*	*z*	*U*_iso_*/*U*_eq_	
Mn	0.77366 (3)	0.606687 (7)	0.227328 (16)	0.00949 (5)	
O1	0.80026 (15)	0.61005 (4)	0.35732 (7)	0.01268 (18)	
O2	0.76671 (14)	0.51776 (3)	0.22257 (7)	0.01220 (16)	
O3	0.71680 (16)	0.69036 (4)	0.65521 (7)	0.0195 (2)	
O4	0.79190 (16)	0.31182 (4)	0.10149 (7)	0.0208 (2)	
O11	0.44142 (13)	0.62058 (4)	0.21616 (7)	0.01380 (19)	
O1S	0.8413 (2)	0.44152 (5)	0.38349 (9)	0.0392 (3)	
H1S	0.8159	0.4732	0.3501	0.047*	
O12	0.11057 (14)	0.61395 (4)	0.21865 (8)	0.0168 (2)	
N1	0.79273 (15)	0.69822 (4)	0.21731 (8)	0.0113 (2)	
N2	0.74229 (17)	0.61290 (4)	0.08940 (8)	0.0104 (2)	
C1	0.76904 (19)	0.65884 (6)	0.41014 (10)	0.0119 (3)	
C2	0.75241 (19)	0.64810 (5)	0.50357 (10)	0.0134 (2)	
H2A	0.7550	0.6067	0.5264	0.016*	
C3	0.73217 (19)	0.69741 (6)	0.56355 (10)	0.0146 (3)	
C4	0.7072 (3)	0.62795 (6)	0.68929 (10)	0.0231 (3)	
H4A	0.5869	0.6070	0.6570	0.035*	
H4B	0.6982	0.6289	0.7560	0.035*	
H4C	0.8301	0.6053	0.6782	0.035*	
C5	0.72696 (19)	0.75947 (6)	0.53186 (11)	0.0157 (3)	
H5A	0.7143	0.7930	0.5732	0.019*	
C6	0.74048 (19)	0.77021 (6)	0.44040 (11)	0.0146 (3)	
H6A	0.7377	0.8119	0.4188	0.018*	
C7	0.75847 (18)	0.72128 (6)	0.37643 (10)	0.0118 (2)	
C8	0.78234 (19)	0.73750 (5)	0.28384 (9)	0.0121 (2)	
H8A	0.7914	0.7805	0.2697	0.014*	
C9	0.8389 (2)	0.71838 (5)	0.12610 (9)	0.0136 (2)	
H9A	0.9868	0.7147	0.1229	0.016*	
H9B	0.7985	0.7624	0.1152	0.016*	
C10	0.7205 (2)	0.67696 (5)	0.05339 (9)	0.0146 (3)	
H10A	0.5752	0.6892	0.0434	0.018*	
H10B	0.7764	0.6803	−0.0062	0.018*	
C11	0.74324 (19)	0.56693 (5)	0.03192 (10)	0.0137 (3)	
H11A	0.7325	0.5767	−0.0322	0.016*	
C12	0.75926 (19)	0.50231 (5)	0.05733 (10)	0.0122 (2)	
C13	0.7634 (2)	0.45918 (5)	−0.01514 (10)	0.0142 (3)	
H13A	0.7566	0.4741	−0.0769	0.017*	
C14	0.7771 (2)	0.39615 (5)	0.00064 (9)	0.0147 (2)	
H14A	0.7825	0.3678	−0.0490	0.018*	
C15	0.78265 (19)	0.37496 (5)	0.09200 (10)	0.0136 (3)	
C16	0.7857 (2)	0.28654 (6)	0.19224 (11)	0.0216 (3)	
H16A	0.9072	0.2998	0.2334	0.032*	
H16B	0.7823	0.2410	0.1887	0.032*	
H16C	0.6636	0.3016	0.2166	0.032*	
C17	0.77871 (19)	0.41586 (5)	0.16587 (9)	0.0126 (2)	
H17A	0.7830	0.4002	0.2271	0.015*	
C18	0.76836 (19)	0.48048 (5)	0.14978 (9)	0.0108 (2)	
C1A	0.2875 (2)	0.59357 (5)	0.23982 (9)	0.0124 (2)	
C2A	0.3177 (2)	0.53384 (6)	0.29512 (12)	0.0230 (3)	
H2AA	0.3698	0.5015	0.2571	0.035*	
H2AB	0.1872	0.5205	0.3134	0.035*	
H2AC	0.4155	0.5410	0.3507	0.035*	
C1S	0.7768 (3)	0.45186 (7)	0.47111 (13)	0.0281 (4)	
H1S1	0.8510	0.4239	0.5169	0.042*	
H1S2	0.6304	0.4437	0.4672	0.042*	
H1S3	0.8044	0.4951	0.4899	0.042*	

### Atomic displacement parameters (Å^2^)

**Table:**

	*U*^11^	*U*^22^	*U*^33^	*U*^12^	*U*^13^	*U*^23^
Mn	0.01095 (8)	0.00751 (7)	0.01000 (8)	0.00012 (7)	0.00128 (8)	0.00023 (7)
O1	0.0164 (4)	0.0094 (4)	0.0124 (5)	0.0016 (3)	0.0022 (4)	−0.0005 (3)
O2	0.0173 (4)	0.0095 (3)	0.0100 (4)	0.0008 (3)	0.0024 (4)	−0.0002 (4)
O3	0.0303 (6)	0.0154 (4)	0.0131 (5)	0.0011 (4)	0.0040 (4)	−0.0029 (4)
O4	0.0377 (6)	0.0095 (4)	0.0156 (5)	0.0000 (4)	0.0044 (5)	−0.0005 (4)
O11	0.0089 (4)	0.0142 (4)	0.0187 (5)	−0.0002 (3)	0.0033 (4)	0.0013 (4)
O1S	0.0710 (9)	0.0293 (6)	0.0181 (6)	0.0173 (6)	0.0087 (7)	0.0048 (5)
O12	0.0117 (4)	0.0169 (4)	0.0222 (6)	−0.0007 (3)	0.0035 (4)	0.0014 (4)
N1	0.0097 (5)	0.0112 (4)	0.0132 (6)	−0.0008 (4)	0.0025 (4)	0.0008 (4)
N2	0.0103 (5)	0.0080 (4)	0.0131 (5)	0.0009 (4)	0.0027 (4)	0.0016 (4)
C1	0.0097 (6)	0.0119 (5)	0.0133 (7)	0.0003 (4)	−0.0014 (5)	−0.0021 (5)
C2	0.0168 (6)	0.0088 (5)	0.0144 (7)	−0.0004 (5)	0.0015 (5)	−0.0013 (5)
C3	0.0143 (6)	0.0173 (6)	0.0121 (7)	0.0002 (5)	0.0012 (5)	−0.0008 (5)
C4	0.0358 (8)	0.0185 (6)	0.0150 (7)	−0.0007 (7)	0.0032 (7)	0.0001 (5)
C5	0.0158 (6)	0.0138 (6)	0.0173 (7)	0.0000 (5)	0.0011 (5)	−0.0056 (5)
C6	0.0135 (6)	0.0104 (5)	0.0196 (8)	0.0001 (5)	0.0010 (5)	−0.0024 (5)
C7	0.0092 (6)	0.0120 (5)	0.0139 (7)	0.0001 (4)	0.0009 (4)	−0.0012 (5)
C8	0.0097 (5)	0.0098 (5)	0.0166 (7)	0.0000 (5)	0.0013 (5)	0.0011 (4)
C9	0.0150 (6)	0.0114 (5)	0.0147 (7)	−0.0022 (5)	0.0033 (5)	0.0024 (5)
C10	0.0193 (6)	0.0106 (5)	0.0140 (6)	0.0018 (5)	0.0022 (6)	0.0048 (5)
C11	0.0147 (6)	0.0151 (5)	0.0111 (6)	0.0007 (5)	0.0013 (5)	0.0018 (5)
C12	0.0138 (6)	0.0112 (5)	0.0114 (6)	0.0004 (5)	0.0010 (5)	−0.0004 (5)
C13	0.0154 (6)	0.0153 (5)	0.0117 (6)	−0.0007 (5)	0.0017 (5)	0.0000 (5)
C14	0.0187 (6)	0.0142 (5)	0.0111 (6)	−0.0014 (5)	0.0019 (5)	−0.0032 (5)
C15	0.0162 (6)	0.0094 (5)	0.0150 (7)	0.0000 (4)	0.0013 (5)	−0.0013 (5)
C16	0.0346 (8)	0.0114 (5)	0.0191 (7)	0.0005 (6)	0.0043 (6)	0.0020 (5)
C17	0.0148 (6)	0.0110 (5)	0.0120 (6)	−0.0002 (5)	0.0014 (5)	−0.0006 (5)
C18	0.0093 (5)	0.0114 (5)	0.0115 (6)	0.0015 (5)	0.0008 (5)	−0.0013 (4)
C1A	0.0146 (5)	0.0116 (5)	0.0108 (6)	0.0041 (5)	0.0005 (5)	−0.0031 (4)
C2A	0.0182 (7)	0.0212 (6)	0.0308 (9)	0.0016 (6)	0.0077 (6)	0.0135 (6)
C1S	0.0338 (9)	0.0290 (8)	0.0218 (9)	0.0001 (7)	0.0048 (7)	0.0003 (7)

### Geometric parameters (Å, °)

**Table:**

Mn—O1	1.8784 (10)	C6—C7	1.4202 (19)
Mn—O2	1.9135 (7)	C6—H6A	0.9500
Mn—N1	1.9786 (9)	C7—C8	1.4208 (19)
Mn—N2	1.9954 (11)	C8—H8A	0.9500
Mn—O11	2.2056 (9)	C9—C10	1.5199 (18)
Mn—O12^i^	2.2571 (9)	C9—H9A	0.9900
O1—C1	1.3327 (15)	C9—H9B	0.9900
O2—C18	1.3297 (15)	C10—H10A	0.9900
O3—C3	1.3597 (17)	C10—H10B	0.9900
O3—C4	1.4349 (16)	C11—C12	1.4380 (16)
O4—C15	1.3650 (14)	C11—H11A	0.9500
O4—C16	1.4339 (17)	C12—C13	1.4075 (18)
O11—C1A	1.2598 (15)	C12—C18	1.4189 (18)
O1S—C1S	1.414 (2)	C13—C14	1.3755 (16)
O1S—H1S	0.8400	C13—H13A	0.9500
O12—C1A	1.2514 (15)	C14—C15	1.4013 (18)
O12—Mn^ii^	2.2571 (9)	C14—H14A	0.9500
N1—C8	1.2935 (16)	C15—C17	1.3919 (18)
N1—C9	1.4663 (17)	C16—H16A	0.9800
N2—C11	1.2954 (16)	C16—H16B	0.9800
N2—C10	1.4741 (14)	C16—H16C	0.9800
C1—C2	1.398 (2)	C17—C18	1.4092 (16)
C1—C7	1.4279 (17)	C17—H17A	0.9500
C2—C3	1.3909 (18)	C1A—C2A	1.5148 (17)
C2—H2A	0.9500	C2A—H2AA	0.9800
C3—C5	1.4109 (17)	C2A—H2AB	0.9800
C4—H4A	0.9800	C2A—H2AC	0.9800
C4—H4B	0.9800	C1S—H1S1	0.9800
C4—H4C	0.9800	C1S—H1S2	0.9800
C5—C6	1.365 (2)	C1S—H1S3	0.9800
C5—H5A	0.9500		
			
O1—Mn—O2	94.22 (4)	N1—C9—C10	107.90 (10)
O1—Mn—N1	92.15 (4)	N1—C9—H9A	110.1
O2—Mn—N1	173.02 (5)	C10—C9—H9A	110.1
O1—Mn—N2	173.93 (3)	N1—C9—H9B	110.1
O2—Mn—N2	91.81 (4)	C10—C9—H9B	110.1
N1—Mn—N2	81.87 (4)	H9A—C9—H9B	108.4
O1—Mn—O11	91.71 (4)	N2—C10—C9	106.41 (10)
O2—Mn—O11	96.53 (4)	N2—C10—H10A	110.4
N1—Mn—O11	86.13 (4)	C9—C10—H10A	110.4
N2—Mn—O11	86.88 (4)	N2—C10—H10B	110.4
O1—Mn—O12^i^	95.22 (4)	C9—C10—H10B	110.4
O2—Mn—O12^i^	94.96 (4)	H10A—C10—H10B	108.6
N1—Mn—O12^i^	81.60 (4)	N2—C11—C12	125.27 (13)
N2—Mn—O12^i^	84.97 (4)	N2—C11—H11A	117.4
O11—Mn—O12^i^	166.11 (3)	C12—C11—H11A	117.4
C1—O1—Mn	127.60 (8)	C13—C12—C18	119.34 (11)
C18—O2—Mn	128.94 (8)	C13—C12—C11	116.87 (12)
C3—O3—C4	117.12 (10)	C18—C12—C11	123.78 (12)
C15—O4—C16	117.64 (10)	C14—C13—C12	122.10 (12)
C1A—O11—Mn	138.82 (8)	C14—C13—H13A	118.9
C1S—O1S—H1S	109.5	C12—C13—H13A	118.9
C1A—O12—Mn^ii^	149.65 (9)	C13—C14—C15	118.14 (12)
C8—N1—C9	121.50 (10)	C13—C14—H14A	120.9
C8—N1—Mn	125.74 (9)	C15—C14—H14A	120.9
C9—N1—Mn	112.57 (8)	O4—C15—C17	123.73 (12)
C11—N2—C10	119.47 (11)	O4—C15—C14	114.49 (11)
C11—N2—Mn	126.15 (9)	C17—C15—C14	121.78 (11)
C10—N2—Mn	114.36 (8)	O4—C16—H16A	109.5
O1—C1—C2	117.98 (11)	O4—C16—H16B	109.5
O1—C1—C7	123.11 (13)	H16A—C16—H16B	109.5
C2—C1—C7	118.88 (12)	O4—C16—H16C	109.5
C3—C2—C1	120.73 (11)	H16A—C16—H16C	109.5
C3—C2—H2A	119.6	H16B—C16—H16C	109.5
C1—C2—H2A	119.6	C15—C17—C18	120.00 (12)
O3—C3—C2	123.82 (12)	C15—C17—H17A	120.0
O3—C3—C5	115.12 (12)	C18—C17—H17A	120.0
C2—C3—C5	121.06 (14)	O2—C18—C17	117.86 (11)
O3—C4—H4A	109.5	O2—C18—C12	123.53 (11)
O3—C4—H4B	109.5	C17—C18—C12	118.61 (11)
H4A—C4—H4B	109.5	O12—C1A—O11	122.45 (11)
O3—C4—H4C	109.5	O12—C1A—C2A	118.79 (12)
H4A—C4—H4C	109.5	O11—C1A—C2A	118.76 (11)
H4B—C4—H4C	109.5	C1A—C2A—H2AA	109.5
C6—C5—C3	118.47 (12)	C1A—C2A—H2AB	109.5
C6—C5—H5A	120.8	H2AA—C2A—H2AB	109.5
C3—C5—H5A	120.8	C1A—C2A—H2AC	109.5
C5—C6—C7	122.39 (12)	H2AA—C2A—H2AC	109.5
C5—C6—H6A	118.8	H2AB—C2A—H2AC	109.5
C7—C6—H6A	118.8	O1S—C1S—H1S1	109.5
C6—C7—C8	117.99 (12)	O1S—C1S—H1S2	109.5
C6—C7—C1	118.41 (13)	H1S1—C1S—H1S2	109.5
C8—C7—C1	123.38 (12)	O1S—C1S—H1S3	109.5
N1—C8—C7	124.97 (11)	H1S1—C1S—H1S3	109.5
N1—C8—H8A	117.5	H1S2—C1S—H1S3	109.5
C7—C8—H8A	117.5		
			
O2—Mn—O1—C1	−163.53 (10)	C5—C6—C7—C8	−176.75 (12)
N1—Mn—O1—C1	19.33 (11)	C5—C6—C7—C1	−1.98 (19)
O11—Mn—O1—C1	−66.86 (10)	O1—C1—C7—C6	−175.17 (12)
O12^i^—Mn—O1—C1	101.09 (10)	C2—C1—C7—C6	2.75 (17)
O1—Mn—O2—C18	−173.04 (11)	O1—C1—C7—C8	−0.71 (19)
N1—Mn—O2—C18	−17.3 (4)	C2—C1—C7—C8	177.21 (13)
O11—Mn—O2—C18	94.75 (11)	C9—N1—C8—C7	−173.48 (12)
O12^i^—Mn—O2—C18	−77.42 (11)	Mn—N1—C8—C7	1.05 (19)
O1—Mn—O11—C1A	−62.12 (14)	C6—C7—C8—N1	−177.21 (12)
O2—Mn—O11—C1A	32.33 (14)	C1—C7—C8—N1	8.3 (2)
N1—Mn—O11—C1A	−154.15 (14)	C8—N1—C9—C10	−145.66 (12)
N2—Mn—O11—C1A	123.80 (14)	Mn—N1—C9—C10	39.15 (12)
O12^i^—Mn—O11—C1A	177.90 (14)	C11—N2—C10—C9	−148.48 (12)
O1—Mn—N1—C8	−11.85 (11)	Mn—N2—C10—C9	30.37 (13)
N2—Mn—N1—C8	167.12 (11)	N1—C9—C10—N2	−43.46 (13)
O11—Mn—N1—C8	79.72 (11)	C10—N2—C11—C12	−178.70 (12)
O12^i^—Mn—N1—C8	−106.81 (11)	Mn—N2—C11—C12	2.60 (19)
O1—Mn—N1—C9	163.10 (9)	N2—C11—C12—C13	−178.25 (12)
N2—Mn—N1—C9	−17.93 (9)	N2—C11—C12—C18	2.5 (2)
O11—Mn—N1—C9	−105.33 (9)	C18—C12—C13—C14	−0.3 (2)
O12^i^—Mn—N1—C9	68.14 (9)	C11—C12—C13—C14	−179.57 (12)
O2—Mn—N2—C11	−6.26 (11)	C12—C13—C14—C15	1.3 (2)
N1—Mn—N2—C11	170.77 (12)	C16—O4—C15—C17	3.60 (18)
O11—Mn—N2—C11	−102.70 (11)	C16—O4—C15—C14	−176.34 (12)
O12^i^—Mn—N2—C11	88.56 (11)	C13—C14—C15—O4	178.69 (12)
O2—Mn—N2—C10	174.99 (9)	C13—C14—C15—C17	−1.3 (2)
N1—Mn—N2—C10	−7.99 (9)	O4—C15—C17—C18	−179.78 (11)
O11—Mn—N2—C10	78.55 (9)	C14—C15—C17—C18	0.2 (2)
O12^i^—Mn—N2—C10	−90.19 (9)	Mn—O2—C18—C17	174.98 (8)
Mn—O1—C1—C2	165.80 (9)	Mn—O2—C18—C12	−5.35 (19)
Mn—O1—C1—C7	−16.26 (17)	C15—C17—C18—O2	−179.44 (11)
O1—C1—C2—C3	176.07 (11)	C15—C17—C18—C12	0.88 (18)
C7—C1—C2—C3	−1.96 (19)	C13—C12—C18—O2	179.53 (11)
C4—O3—C3—C2	−4.39 (19)	C11—C12—C18—O2	−1.3 (2)
C4—O3—C3—C5	175.82 (12)	C13—C12—C18—C17	−0.80 (19)
C1—C2—C3—O3	−179.49 (13)	C11—C12—C18—C17	178.40 (11)
C1—C2—C3—C5	0.3 (2)	Mn^ii^—O12—C1A—O11	−176.52 (12)
O3—C3—C5—C6	−179.64 (12)	Mn^ii^—O12—C1A—C2A	4.1 (3)
C2—C3—C5—C6	0.57 (19)	Mn—O11—C1A—O12	−177.27 (9)
C3—C5—C6—C7	0.3 (2)	Mn—O11—C1A—C2A	2.1 (2)

Symmetry codes: (i) *x*+1, *y*, *z*; (ii) *x*−1, *y*, *z*.

### Hydrogen-bond geometry (Å, °)

**Table:**

*D*—H···*A*	*D*—H	H···*A*	*D*···*A*	*D*—H···*A*
O1S—H1S···O2	0.84	2.08	2.8484 (15)	153
C8—H8A···O11^iii^	0.95	2.51	3.4152 (15)	158
C8—H8A···O12^iii^	0.95	2.63	3.4802 (14)	150
C16—H16B···O3^iv^	0.98	2.52	3.0358 (17)	113

Symmetry codes: (iii) *x*+1/2, −*y*+3/2, *z*; (iv) −*x*+3/2, *y*−1/2, −*z*+1.
